# HIF-2α promotes epithelial-mesenchymal transition through regulating Twist2 binding to the promoter of E-cadherin in pancreatic cancer

**DOI:** 10.1186/s13046-016-0298-y

**Published:** 2016-02-03

**Authors:** Jian Yang, Xu Zhang, Yi Zhang, Dongming Zhu, Lifeng Zhang, Ye Li, Yanbo Zhu, Dechun Li, Jian Zhou

**Affiliations:** Department of General Surgery, The First Affiliated Hospital of Soochow University, 188 Shizi Street, Suzhou, 215006 China; Department of Oncology, The First Affiliated Hospital of Soochow University, Suzhou, 215006 China

**Keywords:** HIF-2α, EMT, Twist, E-cadherin, Pancreatic cancer

## Abstract

**Background:**

Epithelial-mesenchymal transition (EMT) is a dedifferentiation process that mainly involves in mesenchymal marker upregulation, epithelial maker downregulation and cell polarity loss. Related hypoxia factors play a crucial role in EMT, however, it remains few evidence to clarify the role of HIF-2α in EMT in pancreatic cancer.

**Method:**

In this study, we investigated the expression of HIF-2α and E-cadherin by immunohistochemistry in 70 pancreatic cancer patients, as well as the correlation to the clinicopathologic characteristics. Then we regulated the expression of HIF-2α in pancreatic cancer cells to examine the role of HIF-2α on invasion and migration in vitro. Finally, we tested the relation of HIF-2α and EMT related proteins by Western blot and determined whether HIF-2α regulated EMT through Twist regulating the expression of E-cadherin by Chromatin immunoprecipitation (ChIP) assay.

**Results:**

We found that HIF-2α protein was expressed positively in 67.1 % (47/70) of pancreatic cancer tissues and 11.4 % (8/70) of adjacent non-tumor pancreatic tissues, and there was a significant difference in the positive rate of HIF-2α protein between two groups (*χ*2 = 45.549, *P* < 0.05). In addition, the staining for HIF-2α was correlated with tumor differentiation (*P* < 0.05), clinical stage (*P* < 0.05) and lymph node metastasis (*P* < 0.05), while E-cadherin expression was only correlated with lymph node metastasis (*P* < 0.05). HIF-2α promoted cell migration, invasion in vitro, and regulated the expression of E-cadherin and MMPs, which are critical to EMT. Our further ChIP assay suggested that only Twist2 could bind to the promoter of E-cadherin in -714 bp region site, but there is no positive binding capacity in -295 bp promoter region site of E-cadherin. Clinical tissues IHC staining showed that Twist2 and E-cadherin expression had an obviously negative correlation in pancreatic cancer. Nevertheless, it had no obvious correlation between Twist1 and E-cadherin.

**Conclusion:**

These findings indicated that HIF-2α promotes EMT in pancreatic cancer by regulating Twist2 binding to the promoter of E-cadherin, which meant that HIF-2α and this pathway may be effective therapeutic targets for pancreatic cancer.

## Background

Pancreatic cancer is a solid malignancy which is generally characterized by a poor prognosis. The radical resection of pancreatic tumors, especially in the stage of precursor lesions, may be the only hope for cure [1]. However, even after surgical resection, the 5-year survival is only 20 % due to its high recurrence rate [2], in addition, radiotherapy and chemotherapy obtain little benefit [3]. Vascular invasion and distant metastasis are the critical features in the aggressive phenotype of pancreatic cancer.

As solid tumors growing, their microenviromental condition becomes gradually hypoxic. Under conditions of hypoxia, a signaling pathway involving a crucial oxygen response regulator, defined hypoxia-inducible factor (HIF), is turned on [4]. Misregulation of HIF protein, especially HIF-1α and HIF-2α, is correlation with tumor development and metastasis [5]. Substantial experiments were carried out to determine the role and mechanism of HIF-1α in various tumors. In contrast with HIF-1α, which is expressed in most metazoan species, the expression of HIF-2α is observed in certain cell types of vertebrate species [6]. Indeed, HIF-2α has been proved to play an important role in many aspects of digestive cancers, containing proliferation, angiogenesis metabolism, metastasis and resistance to chemotherapy [7].

Epithelial-mesenchymal transition (EMT) is a dedifferentiation process, which plays an integral role in tumor progression [8]. In the process of EMT, cells acquired mesenchymal characteristics and lost epithelial phenotypes, mainly involved in mesenchymal marker upregulation, epithelial maker downregulation and cell polarity loss [8, 9]. Loss of E-cadherin plays a key role in the EMT differentiation process and leads to increase cellular motility and invasion. As a main EMT-mediated transcription factor, twist reportedly contributes to cadherin switching. Interestingly, twist is a member of the basic helix-loop-helix (bHLH) transcription factor family and structural similarity with HIF at the bHLH [10, 11]. The function of HIF and Twist may have some similarity. Research has been demonstrated that Twist is correlated with metastasis of multiple malignant tumors of epithelial origin [12] and involves in the regulation of EMT [10, 13].

Related hypoxia factors play a crucial role in EMT [14], however, there is little evidence to clarify the role of HIF-2α in EMT in pancreatic cancer. In this study, we examined the expression of HIF-2α and E-cadherin in pancreatic cancer, as well as the correlation to the clinicopathologic characteristics. Then we investigated the role of HIF-2α in EMT process in pancreatic cancer cells. Finally, we tested the relation of HIF-2α and EMT related proteins and determined whether HIF-2α regulated EMT through Twist regulating the expression of E-cadherin.

## Methods

### Clinical samples

Tumor tissues of 70 patients were obtained from the First Affiliated Hospital of Soochow University from 2011 to 2013. Formalin-fixed tumor tissues were used for immunohistochemistry (IHC), including tumor samples and matching adjacent non-tumor tissues. Detailed clinicopathological data were recorded, including each patient’s age, gender, tumor size, tumor differentiation, and lymph node metastasis, and tumor clinical stages were classified according to the UICC staging system. None of the patients received chemotherapy, radiotherapy or immunotherapy before surgery. All the samples were obtained following patient consent and approval by the Ethics Committee of Soochow University.

### Expression plasmids and HIF-2α gene silencing

Full-length HIF-2α complementary DNA (cDNA) was amplified by normal human embryo cDNA, digested with XhoI/EcoRIand subcloned into pcDNA3.1 vectors (OE-HIF-2α). The empty pcDNA3.1 vectors served as a negative control (Vector). The small interfering RNA (siRNA) was constructed by GeneChem Co., Ltd. (Shanghai, China). The siRNA sequence targeting HIF-2α (si-HIF-2α) was 5′-GCAAATGTACCCAATGATA-3′, as confirmed by sequencing. A nonspecific scrambled siRNA sequence (si-Scramble) was used as a negative control (target sequence 5′- GTTCTCCGAACGTGTCACGT-3′).

### Cell culture and transfection

The pancreatic cancer cell lines of AsPC-1, CaPan-2, PaTu8988, SW1990, BXPC-3 were obtained from the Chinese Academy of Sciences (Shanghai, China). The cells were maintained with DMEM (HyClone, Shanghai, China) supplemented with 10 % fetal bovine serum (FBS, HyClone, Shanghai, China) and cultured at 37 °C in a humidified atmosphere containing 5 % CO2. SW1990 and AsPC-1 cell expressing OE-HIF-2α or si-HIF-2α were performed by Lipofectamine 2000 (Invitrogen, CA, USA).

### Western blot

Cells were collected and lysed in lysis buffer on ice. Total proteins were separated by 10 % SDS-PAGE and blotted on PVDF membrane. Membranes were blocked with 10 % non-fat milk powder at room temperature for 2 h and incubated with primary antibodies: anti-HIF2α (1:200), anti-VE-cadherin (1:1000), anti-MMP2 (1:1000), anti-MMP9 (1:5000), anti-Twist1 (1:200), anti-Twist2 (1:50) (all from Abcam, Cambridge, UK) and anti-GAPDH (1:1000, Santa Cruz Biotechnology, CA, USA), at 4̊C overnight. After three washes, the membranes were incubated with a horseradish peroxidase-conjugated goat anti-mouse IgG (1:2000; Santa Cruz Biotechnology). Reactive bands were detected using ECL western blotting detection reagent (GE Healthcare, USA).

### Wound healing assay

Cells from each group were incubated in 6-well plates. A small wound area was made in the confluent monolayer with a 200 μl pipette tip in a lengthwise stripe. The cells were washed twice with PBS and incubated at 37̊C. The speed of wound closure was monitored after 24 and 48 h by measuring the ratio of the distance of the wound at 0 h. Wound width was measured at 100× magnifiation using a microscope (Leica Microsystems, Mannheim, Germany). Each experiment was performed in triplicate.

### Cell invasion assay

Cell invasion was performed in Transwell cell culture chambers with 8 μm pores (Corning, NY,USA). The inserts in the membrane filter were coated with Matrigel on the upper surface. Cells at concentration of 5 × 10^5^/ml resuspended in serum-free DMEM were placed on the upper chamber, while the lower chamber was filled with DMEM with 10%FBS. After incubation at 37 °C for 48 h, the cells on the upper surface of the filter were removed with a cotton swab. Invading cells at the bottom of Matrigel were fixed in methanol and stained with 0.1 % crystal violet. The number of invading cells was counted under a microscope at 200 × magnification of 5 random fields per well. Each experiment was performed in triplicate.

### Chromatin immunoprecipitation assay

Chromatin immunoprecipitation (ChIP) assay was performed using a ChIP assay kit (Upstate Biotechnology, LP, USA) as described by the manufacturer. AsPC-1 cells were lysed, and the immunoprecipitation was performed with anti-Twist1 polyclonal antibody (Santa Cruz Biotechnology, CA, USA), anti-Twist2 monoclonal antibody (Abcam, Cambridge, UK) or mouse immunoglobulin G (IgG; negative control). Following the wash, the antibody-protein-DNA complex was eluted from the beads and reversed cross-link incubation. After removed protein and RNA, purified DNA was subjected to polymerase chain reaction (PCR) with primers specific for human E-cadehrin promoter. The primers for PCR were as follows: P1: F: 5′- GAACCCAAGAGGCGAAGG-3′ and R: 5′-GGTGCTGGACATTGAAGATTACT − 3′(154 bp); P2: F: 5′-GCCAGGATGGTCTCAATCTC-3′ and R: 5′-CTCCCTATGCTGTTGTGGG-3′(194 bp).

### Immunohistochemistry (IHC)

Serial sections (4 μm) subjected to immunohistological staining were fixed with freshly prepared 3 % H_2_O_2_ with 0.1 % sodium azide to quench endogenous peroxidase and then treated with antigen retrieval solution for 15 min. After placing in blocking reagent for 15 min, the sections were incubated in primary anti-HFI-2α (1:500, Abcam), anti-E-cadherin (1:1000, Abcam), anti-Twist1 (1:500, Abcam) and anti-Twist2 (1:350, Abcam) monoclonal antibody overnight at 4 °C, followed by incubation with the secondary antibody and Extravidin-conjugated horseradish peroxidase. The staining intensity was scored as: 0 (<10 %), 1 (10–50 %), 2 (>50 %). The final score was calculated by multiplication of the intensity score the quantity score. A score ≥ 2 was considered to represent positive expression.

### Statistical analysis

All data in the study were evaluated with SPSS version 18.0 software. Data were presented as mean ± SD. Continuous variables were compared one-way analysis of variance (oneway ANOVA) and categorical variable were compared by Chi-square test. Correlation analysis was performed using Spearman analysis. Difference was considered significant at values of *P* < 0.05.

## Results

### Expression of HIF-2α and E-cadherin in pancreatic cancer

**Fig. 1:**
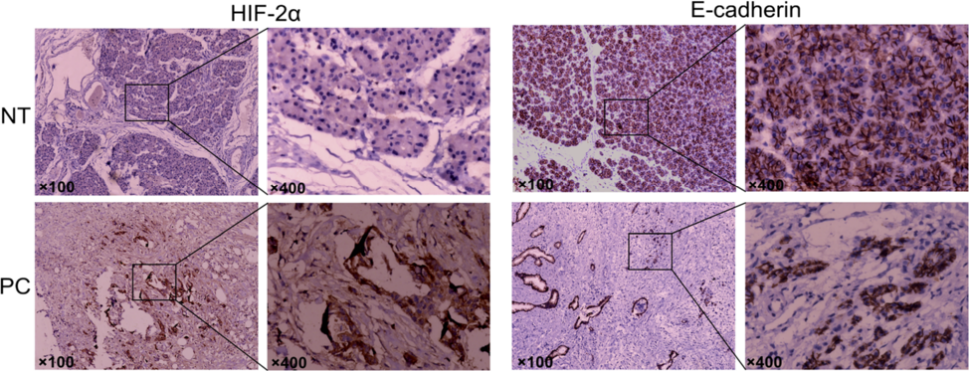
Expression of HIF-2α and E-cadherin in pancreatic cancer and adjacent non-tumor tissues. Original magnification × 100 or 400. Images are representative of three independent experiments

To investigate the roles of HIF-2α and E-cadherin in the progression of pancreatic cancer, we firstly detected the expression of HIF-2α and E-cadherin proteins in 70 pancreatic cancer tissues and matching adjacent non-tumor tissues by IHC staining. In our immunostaining results, the location of HIF-2α protein was observed mainly in the cytoplasm and nucleus, while the staining for E-cadherin was confined to the cytomembrane (Fig. 1). HIF-2α protein was expressed positively in 67.1 % (47/70) of pancreatic cancer tissues and 11.4 % (8/70) of adjacent non-tumor pancreatic tissues. There was a significant difference in the positive rate of HIF-2α protein between the pancreatic cancer tissues group and non-tumor tissues group (*χ*2 = 45.549, *P* < 0.05). Whereas, the expression of E-cadherin protein was significantly lower in pancreatic tumor tissues (21/70) than in non-tumor tissues (43/70), showing a significant difference (*χ*2 = 13.931, *P* < 0.05).

We further investigated whether expression of HIF-2α and E-cadherin was correlated with clinicopathological characteristics of pancreatic cancer patients. As shown in Table 1, HIF-2α expression was correlated with tumor differentiation (*χ*2 = 6.921, *P* = 0.026), clinical stage (*χ*2 = 6.460, *P* = 0.017) and lymph node metastasis (*χ*2 = 5.250, *P* = 0.040). However, the staining for HIF-2α had no significant association with gender, age, tumor location, tumor size (*P* > 0.05). These results indicated that the overexpression of HIF-2α might be correlated with poor differentiation and advanced clinical stage of pancreatic cancer. On the other hand, the staining for E-cadherin was obviously only associated with lymph node metastasis (*χ*2 = 8.221, *P* = 0.006). In Table 2, we found that expression of HIF-2α was negatively related to E-cadherin in pancreatic cancer tissues (*r* = −0.394, *P* < 0.05).

**Table 1:** Expression of HIF-2α and E-cadherin, and the relation with the clinicopathologic features in pancreatic carcinoma

Variables		HIF-2α			E-cadherin		
	No.	Positive	Negative	*P*-value	Positive	Negative	*P*-value
Gender				0.306			0.424
Male	42	26	16		13	29	
Female	28	21	7		6	22	
Age (years)				1.000			0.107
≤ 65	41	28	13		8	33	
> 65	29	19	10		11	18	
Tumor location				0.569			0.226
Head	52	36	16		12	40	
Body and tail	18	11	7		7	11	
Tumor size (cm)				0.122			0.414
≤ 2	27	15	12		9	18	
> 2	43	32	11		10	33	
Differentiation				0.026			0.784
Well	8	3	5		3	5	
Moderate	17	9	8		5	12	
Poor	45	35	10		11	34	
Clinical stage				0.017			0.579
I	25	12	13		8	17	
II	45	35	10		11	34	
Lymph node Metastasis							
Yes	38	30	8	0.040	5	33	0.006
No	32	17	15		14	18

**Table 2:** The relationship of HIF-2α and E-cadherin in pancreatic cancer tissues

Variables	HIF-2α		r	*P*-value
	Positive	Negative		
E-cadherin				
Positive	7	12	−0.394	0.002
Negative	40	11		

### UP-regulation and down-regulation of HIF-2α in pancreatic cell lines

**Fig. 2:**
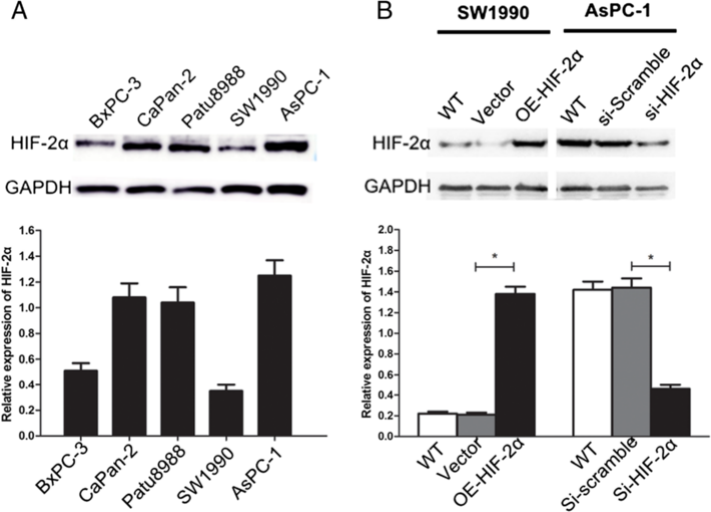
Expression of HIF-2α in pancreatic cell lines and regulating HIF-2α in AsPC-1 and SW1990 cells. **a** Relative expression of HIF-2α protein in pancreatic cancer cell lines (BXPC-3, CaPan-2, PaTu8988, SW1990 and AsPC-1) was measured by Western blot. **b** Expression of ectopic expression and knockdown of HIF-2α in SW1990 or AsPC-1 cells by Western blot. Images are representative of three independent experiments. *P < 0.05

We detected and compared the expression level of HIF-2α protein in five pancreatic cell lines, including BxPc-3, CaPan-2, Patu8988, SW1990 and AsPC-1 by Western Blot. From the results, we found that AsPC-1 cells had a highest expression of HIF-2α in contrast with the SW1990 cells, which expressed a low level (Fig. 2a). To investigate whether HIF-2α contributes to EMT, we first established AsPC-1 cells with silencing of HIF-2α expression by siRNA, on the contrary, SW1990 cells were transfected with HIF-2α cDNA to up-regulate HIF-2α expression. As shown in Fig. 2b, the expression of HIF-2α was significant up-regulated in SW1990 cells following transfection with OE-HIF-2α cells (*P* < 0.05), while the expression of HIF-2α was significant down-regulated in AsPC-1 cells following transfection with si-HIF-2α cells (*P* < 0.05).

### HIF-2α promotes cell migration, invasion in vitro

**Fig. 3:**
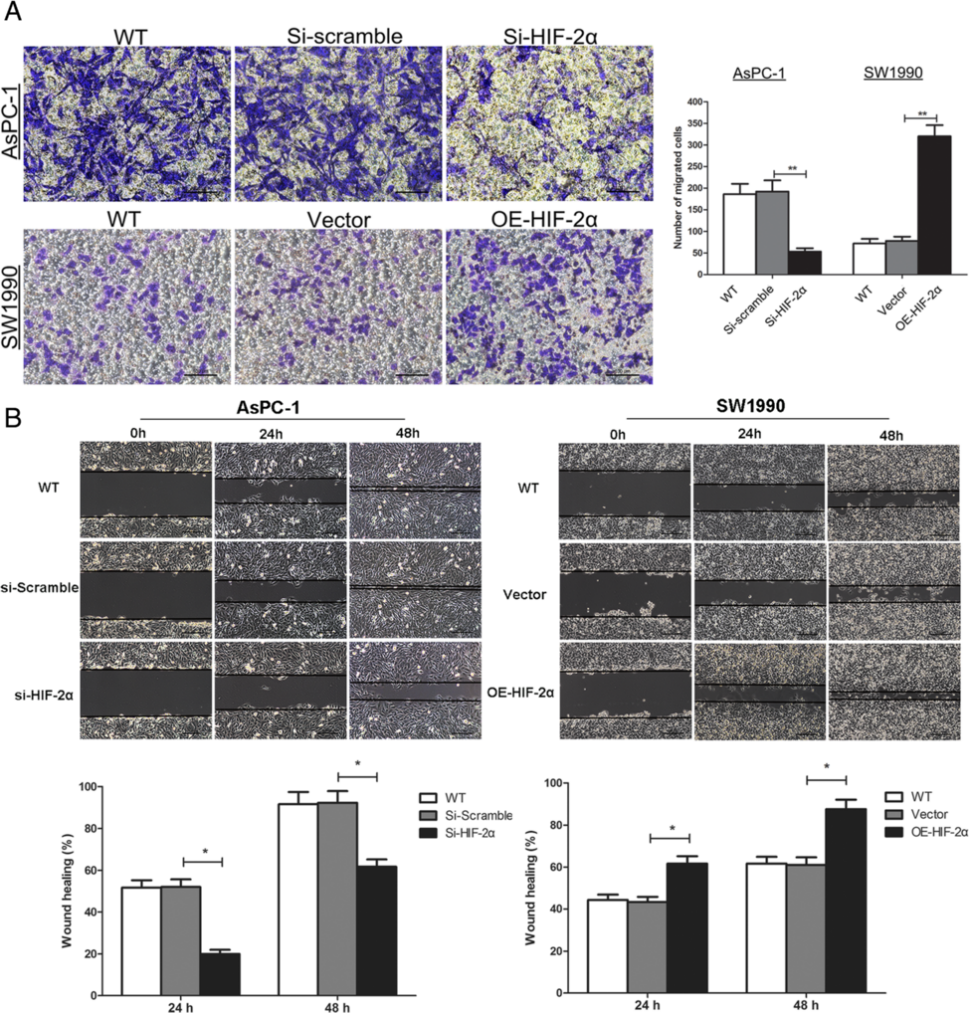
HIF-2α promoted cell migration and invasion. **a** Cell invasion was detected by the Transwell assay. Representative images of cell invasion captured under an inverted microscope (original magnifiation, ×200). The data represent the means ± SD of 5 experiments. **P < 0.01. **b** Cell migration was detected by the wound scrape assay. Representative images of cell migration in the wound scrape model at 0, 24 h and 48 h are shown; original magnifiation, ×100. The data represent the means ± SD of three experiments. *P < 0.05

EMT is considered to be associated with cell migration and invasion among tumor cells. We investigated the cell migration and invasion ability via the wound healing assay and transwell systems after performing HIF-2α ectopic transfection or HIF-2α knockdown, respectively. In the transwell assay, about 3-fold a decrease in cells passed Matrigel was observed in si-HIF-2α group compared with si-Scramble groups (*P* < 0.01). We also found a 5-fold increase in OE-HIF-2α group than Vector group (*P* < 0.01) (Fig. 3a). Similar results showed in the wound healing assay that si-HIF-2α cells conducted relatively slower migration towards the wound space compared with si-Scramble cells (*P* < 0.05) (Fig. 3b). These results showed that HIF-2α played an important role in the progress of pancreatic cancer cells in vitro, and perhaps participated in the EMT process through increasing the ability of cell migration and invasion.

### Effect of HIF-2α on the EMT related proteins

**Fig. 4:**
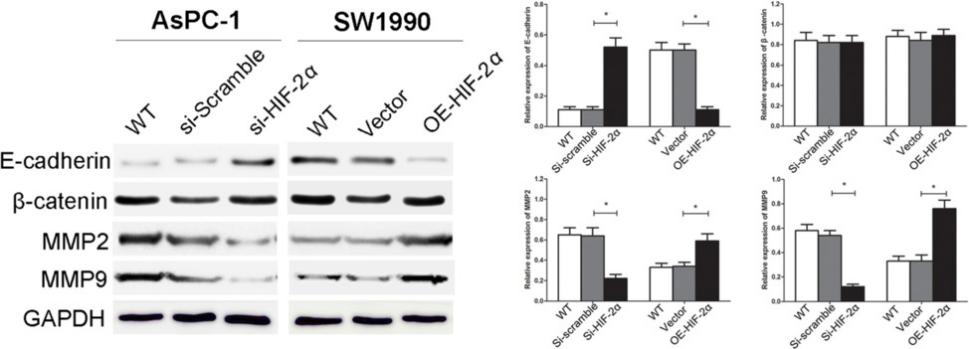
Effect of HIF-2α on expression of EMT-related proteins. E-cadherin, β-catenin, MMP2 and MMP9 was examined in AsPC-1 cells silencing of HIF-2α expression and SW1990 cells transfected with HIF-2α cDNA by Western blot. Images are representative of three independent experiments. *P < 0.05

To understand the interval molecular mechanism of HIF-2α involving in EMT, we investigated the expression levels of EMT related proteins after regulating HIF-2α by Western Blot. As shown in Fig. 4, a significantly increased expression of E-cadherin was observed in si-HIF-2α AsPC-1 cells compared with si-Scramble cells (*P* < 0.05). In SW1990 cells, after up-regulating HIF-2α through transfecting HIF-2α cDNA, the results of which showed a decrease of the expression of E-cadherin (*P* < 0.05). It had little effect on β-catenin when regulated HIF-2α in these two cell lines (*P* > 0.05). To further determine whether HIF-2α affected MMPs expression in pancreatic cancer cells, we analyzed the expression of MMPs in both AsPC-1 and SW1990 cells. After down-regulating HIF-2α, expression level of MMP2 and MMP9 was reduced in si-HIF-2α group compared with si-Scramble groups in AsPC-1 cells (*P* < 0.05, respectively). Similarly, findings were shown in SW1990 cells, OE-HIF-2α group expressed higher MMP2 and MMP9 level than the negative Vector group (*P* < 0.05, respectively). These results demonstrated that HIF-2α regulated the expression of E-cadherin and MMPs, which were critical to EMT.

### HIF-2α promotes EMT through Twist binding the promoter region of E-cadherin

**Fig. 5:**
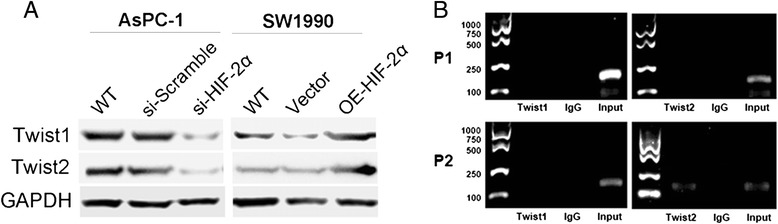
HIF-2α promotes EMT through Twist binding to the promoter region of E-cadherin. **a** Twist1 and Twist2 expressions in response to up-regulation or silencing of HIF-2α in pancreatic cancer cells were detected by Western blot. **b** ChIP of Twist1 and Twist2 interactions with the E-cadherin promoter. Bands are PCR products targeting P1 and P2 of the E-cadherin promoter. The specific anti-Twist1, anti-Twist2 or control normal mouse IgG was used for immunoprecipitations, whereas genomic DNA was used as the input control

Twist is identified as a main transcription factor associated with EMT process [15]. We firstly investigated whether HIF-2α affected the expression of Twist1 and Twist2. As shown in Fig. 5a, silencing of HIF-2α in cells could decrease the level of Twist1 and Twist2 proteins (*P* < 0.05, respectively), and similar results were shown in up-regulating HIF-2α (*P* < 0.05, respectively). It suggested that HIF-2α could regulate the expression of Twist1 and Twist2. In the process of EMT, E-cadherin plays a key role as a regulator in intercellular adhesion. The promoter region of the E-cadherin gene was analyzed by Patch software to identify potential binding sites for transcription factors. Two potential Twist protein binding sites, separately designated P1 (−295 bp) and P2 (−714 bp) were identified by the Patch database of transcription factor binding sites (Table 3). To test whether Twist1 and Twist2 could bind to the promoter region of E-cadherin, we performed a ChIP assay on AsPC-1 cells, which was overexpression of Twist1 and Twist2. The Twist2 antibody specifically immunoprecipitated a Twist2-DNA complex in the correlated region of the E-cadherin promoter, and the binding of Twist2 and P2 regions was demonstrated by PCR performed with relevant specific primers (Fig. 5b). But the results showed Twist2 had no positive binding capacity to the P1 region site of E-cadherin. However, there were no obviously results to demonstrate the Twist1 had binding capacity to the transcription region of E-cadherin in both P1 and P2 binding sites through the ChIP assay. These results indicated that Twist2 was directly bound to E-cadherin promoter through P2 region, but not P1 region.

**Table 3:** Predict potential Twist binding sites in the promotor region of E-cadherin gene (Patch software)

Twist binding site	Position	Sequence
P1	−310 bp to -291 bp (+)	gtgt**CAAATG**cttagcacag
P2	−729 bp to -710 bp (−)	gaaa**CATTTG**tcatttaatt

### The relationship of Twist1/2 and E-cadherin in clinical tissues

**Fig. 6:**
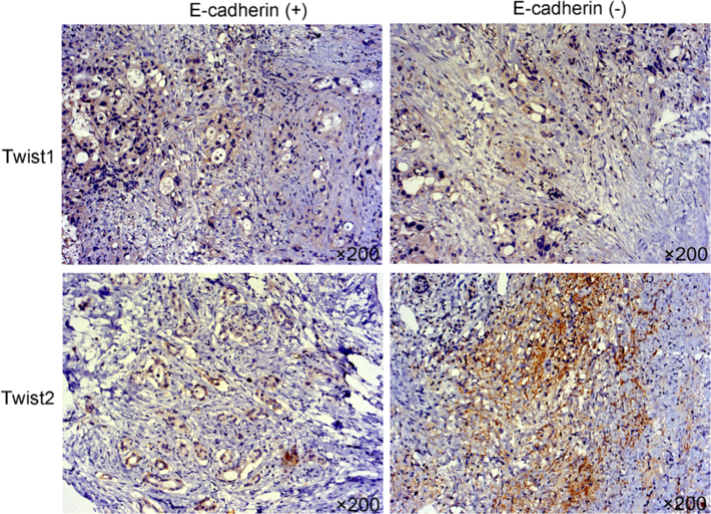
Representative immunohistochemical staining of Twist1 and Twist2 in the two groups of pancreatic cancer tissues with E-cadherin positive or E-cadherin negative expression. Original magnification × 200. Images are representative of three independent experiments

To obtain clinical evidence of the correction between Twist1, Twist2 and E-cadherin, we tested the expression of Twist1 and Twist2 in the above-mentioned 70 pancreatic cancer tissues by IHC staining. As shown in Fig. 6, the results showed that Twist1 and Twist2 expressions were located in the cytoplasm of pancreatic cancer cells. The difference in Twist1 expression was no statistically significant between E-cadherin positive expression and negative expression (*P* > 0.05). While, the E-cadherin negative group expressed a higher level of Tiwst2 compared with the positive group (*P* < 0.05). In Table 4, our results showed that Twist2 and E-cadherin expression had an obviously negative correlation in pancreatic cancer tissues (*r* = − 0.417, *P* < 0.05), however, it had no obvious correlation between Twist1 and E-cadherin (*r* = − 0.114, *P* > 0.05).

**Table 4:** Correlation of Twist1/2 and E-cadherin in pancreatic cancer tissues

	E-cadherin			
Variables	Positive	Negative	r	*P*-value
Twist1			−0.114	0.341
Positive	9	27		
Negative	10	24		
Twist2			−0.417	0.001
Positive	7	41		
Negative	12	10		

## Discussion

As a most deadly gastrointestinal carcinoma, pancreatic cancer leads to a hypoxic environment because of its rapidly growth and plentiful oxygen demand. HIF-2α is confined to the cell nucleus and expresses only under the condition of hypoxic stimulation. A better understanding the molecular mechanism of HIF-2α may be benefit to explore new promising therapeutic strategies for the treatment of pancreatic cancer. In our present study, we examined the expression of HIF-2α in 70 matched clinical pancreatic cancer tissues. The results showed that HIF-2α was overexpressed in pancreatic cancer tissues, and HIF-2α expression was correlated with poor differentiation, advanced clinical stage and lymph node metastasis. It suggested that overexpression of HIF-2α was frequently detected in patients with poor pathological characteristics.

Hypoxia is a common phenomenon in many tumors, especially in most types of human tumors, including breast, colon, ovarian, pancreatic, prostate, renal and hepatocellular cancers [7, 16]. HIF-1α and HIF-2α are key transcription factors in tumor development and only accumulate in hypoxic tumors [17, 18]. HIF-2α, as an important hypoxia-related factor, regulates the adaptive response to decreased O_2_ availability at the cellular and organismal level [7]. Previous report shows that HIF-2α is involved in the invasion and metastasis of gastric cancer cells under hypoxia [19]. In our study, depletion of HIF-2α expression obviously inhibited the migration and invasion of AsPC-1 cells, and the opposite effect was shown in upregulation of HIF-2α in SW1990 cells. Thus, it was plausible to consider that HIF-2α promoted cell invasion and migration in pancreatic cancer.

EMT plays an important role in the development of tissues during embryogenesis, however, similar phenomena was found in pathological processes, including cancer [20]. As the first stage of invasion and metastasis, EMT was reported involving in the development of many solid cancers, including gastric cancer [21], colon cancer [22] and breast cancer [23]. Metastasis is a complex process, and it represents products of a multistep cell-biological process termed the invasion-metastasis cascade, which involves spread of cancer cells to distant organs and adaptation to their microenvironments [24]. The mechanism between tumor cell plasticity and the EMT process may be the same and may thus have a similar relevance [25].

Several pivotal proteins are necessary for the occurrence and progress of EMT in various cancers, among which E-cadherin is the most critical regulator. E-cadherin is a typical epithelial marker, and loss of E-cadherin is an important characteristic of occurrence of EMT [26], the reason for this program is that loss of E-cadherin expression confuses cell polarity and also decreases the stability in epithelial cells. The expression of E-cadherin is regularly lost or decreased in advanced tumors and it is possibly linked to a higher incidence of metastasis and recurrence [27]. Our present findings revealed that pancreatic cancer expressed obviously lower E-cadherin than matched adjacent non-tumor tissues and E-cadherin expression was negatively correlated with lymph node metastasis. It indicates that decreased E-cadherin level contributes to reduce the combining capacity and stability among pancreatic cancer cells, thus, it provides convenience for cells to metastasis. In our study, HIF-2α may induce EMT via decreasing the expression of E-cadherin. Meanwhile our study showed that overexpression of HIF-2α enhanced the expression of MMP2 and MMP9, which are closely related to tumor metastasis, are also significant to EMT [28]. This result further confirmed that HIF-2α promoted EMT in pancreatic cancer. It seems reasonable that HIF-2α takes part in the development of pancreatic cancer through EMT to promote the invasion and metastasis of pancreatic cancer.

Twist1 and Twist2, as the main EMT-mediated process regulators, express high structural homology, and gene deletion tests have proved that two proteins share some similar effects and functions, such as their role in tumor progression and metastasis and their regulation of hematological malignancies [29, 30]. Twist can reduce the expression of E-cadherin through binding to the two bipartite E-box motifs within E-cadherin promoter and inhibiting its transcription [31], suggesting that Twist contributes to metastasis by promoting EMT. However, the mechanism of HIF-2α, Twist1/2 and E-cadherin is still poorly understood. In the present study, we found that Twist1, Twist2 and E-cadherin were regulated by HIF-2α in pancreatic cancer cells. Our further ChIP assay suggested that only Twist2 could bind to the promoter of E-cadherin in -714 bp region site, but there was no positive binding capacity in -295 bp promoter region site of E-cadherin. Twist1 had no similar effect on above-mentioned promoter region site of E-cadherin. Clinical tissues IHC staining showed that Twist2 and E-cadherin expression had an obviously negative correlation in pancreatic cancer tissues, however, it had no obvious correlation between Twist1 and E-cadherin. While Sun et.al indicated that Twist1 contributed to EMT process through down-regulation of E-cadherin expression in hepatocellular carcinoma (HCC) [25]. Those studies along with our findings indicate that the function of Twist1/2 and E-cadherin in cancer progression depends on different tumor types. The accurate mechanism of how Twist1/2 affects the promoter capacity of E-cadherin is remained to be characterized.

## Conclusion

In conclusion, our study demonstrated that HIF-2α was overexpressed in pancreatic cancer and associated with poor pathological characteristics. Our findings indicate that HIF-2α promotes EMT in vitro, at least in part by regulating Twist2/E-cadherin pathway. And whether blockage of HIF-2α may prove to be an effective therapeutic strategy in pancreatic cancer deserved further exploration.

### Ethics approval and consent to participate

This study has been approved by the ethics committee of the First Affiliated Hospital of Soochow University. Patients who were enrolled in this study had signed the informed consent.

## Abbreviations

bHLH: is short for basic helix-loop-helix
ChIP: is short for Chromatin immunoprecipitation
EMT: is short for Epithelial-mesenchymal transition
HIF-2α: is short for hypoxia-inducible factor-2α
IHC: is short for immunohistochemistry
